# Minute-Made, High-Efficiency Nanostructured Bi_2_Te_3_ via High-Throughput Green Solution Chemical Synthesis

**DOI:** 10.3390/nano11082053

**Published:** 2021-08-12

**Authors:** Bejan Hamawandi, Hazal Batili, Moon Paul, Sedat Ballikaya, Nuzhet I. Kilic, Rafal Szukiewicz, Maciej Kuchowicz, Mats Johnsson, Muhammet S. Toprak

**Affiliations:** 1 Department of Applied Physics, KTH Royal Institute of Technology, SE-106 91 Stockholm, Sweden; batili@kth.se (H.B.); moonp@kth.se (M.P.); nikilic@kth.se (N.I.K.); 2 Department of Physics, University of Istanbul, Istanbul 34135, Turkey; ballikaya@istanbul.edu.tr; 3 Institute of Experimental Physics, University of Wroclaw, Maxa Borna 9, 50-204 Wroclaw, Poland; szuszu@ifd.uni.wroc.pl (R.S.); macko20@ifd.uni.wroc.pl (M.K.); 4 Department of Materials and Environmental Chemistry, Stockholm University, SE-106 91 Stockholm, Sweden; mats.johnsson@mmk.su.se

**Keywords:** nanochemistry, bismuth telluride, thermoelectric, nanoparticles, colloidal synthesis, green chemistry, thermoelectric figure-of-merit, ZT, nanocharacterization, thermal conductivity

## Abstract

Scalable synthetic strategies for high-quality and reproducible thermoelectric (TE) materials is an essential step for advancing the TE technology. We present here very rapid and effective methods for the synthesis of nanostructured bismuth telluride materials with promising TE performance. The methodology is based on an effective volume heating using microwaves, leading to highly crystalline nanostructured powders, in a reaction duration of two minutes. As the solvents, we demonstrate that water with a high dielectric constant is as good a solvent as ethylene glycol (EG) for the synthetic process, providing a greener reaction media. Crystal structure, crystallinity, morphology, microstructure and surface chemistry of these materials were evaluated using XRD, SEM/TEM, XPS and zeta potential characterization techniques. Nanostructured particles with hexagonal platelet morphology were observed in both systems. Surfaces show various degrees of oxidation, and signatures of the precursors used. Thermoelectric transport properties were evaluated using electrical conductivity, Seebeck coefficient and thermal conductivity measurements to estimate the TE figure-of-merit, ZT. Low thermal conductivity values were obtained, mainly due to the increased density of boundaries via materials nanostructuring. The estimated ZT values of 0.8–0.9 was reached in the 300–375 K temperature range for the hydrothermally synthesized sample, while 0.9–1 was reached in the 425–525 K temperature range for the polyol (EG) sample. Considering the energy and time efficiency of the synthetic processes developed in this work, these are rather promising ZT values paving the way for a wider impact of these strategic materials with a minimum environmental impact.

## 1. Introduction

Our heavy dependence on non-renewable sources have led to a global energy crisis and as the consumption of these sources has increased, the prospect of their availability for a long term has reduced [1]. Certainly, to meet future energy challenges and climate change it is of paramount importance to develop renewable, and sustainable energy solutions. Thermoelectric (TE) materials have received significant attention because of their ability to convert waste heat to electrical power directly [2]. They can be used to harvest low- and high-grade waste-heat effectively, which requires the selection of proper material for the intended temperature range. As TE materials can be used for power generation, they can also be used for cooling or thermal management. TE devices have attracted significant interest because of their potential applications in energy harvesting, waste heat recovery in industries and transportation systems, chip cooling, solar cells, temperature sensing, military and space exploration [3,4,5,6,7]. Conventional TE devices are made of compact solid-state materials without any moving parts, which gives them a high operational reliability and makes them scalable [8,9]. The performance of the TE devices is strongly dependant on the efficiency of the TE materials [10], which has been formulated as a dimensionless quantity, the TE figure of merit (ZT) and is composed of electrical and thermal transport terms. Specifically, it is defined as *ZT* = S^2^σT/κ (where S is the Seebeck coefficient, σ is the electrical conductivity, T is the absolute temperature, and κ is the thermal conductivity, which is the sum of electronic (κ_el_) and lattice (κ_lat_) components) [11]. The desired qualities in good TEs are high σ, large S accompanied by a low κ. Nanostructuring has become an important approach in the field of TE for controlling the thermal conductivity κ independent from the electrical conductivity σ [12]. Considerable amount of research has shown that low-dimensional structures could significantly improve the TE performance due to quantum confinement and scattering of a broad range of heat carrier phonons [13,14]. This concept yielded some strategies for the enhancement of ZT by reducing κ, while maintaining σ, by taking advantage of different phonon and electron mean free paths (MFPs). The κ is more sensitive to nanostructuring than the σ due to the longer MFP of the thermal phonons than that of the charge carriers [15]. Since in nanostructures there is a large density of interface, the phonons are scattered more effectively than the electrons [16]. Thus, due to the phonon blocking effect, the κ_lat_ reduces in nanostructures while not affecting the mobility and σ significantly. The enhanced ZT in TE materials has been predicted, or experimentally demonstrated for nanostructures like quantum dots [17], thin film superlattices [18], 1D structures–nanowires [19], nanotubes [20], and 2D quantum wells [21]. Microstructure engineering through targeted nanostructure/matrix crystallographic alignment was shown to create endotaxially placed nanoscale precipitates within the matrix, leading to very low thermal conductivity values [22]. Well-controlled nanoscale-shaped interfaces and oriented nanocrystals were also demonstrated to reduce κ beyond the amorphous limit, while preserving bulk-like σ [12]. Rather promising ZT was achieved in roughened Si nanowires, which has been mainly attributed to efficient scattering throughout the phonon spectrum by the introduction of nanostructures at different length scales (diameter, roughness and point defects) [23].

The most common n-type TE material for room temperature operations is Bi_2_Te_3_. A survey on the different methods of its synthesis and the variety of morphologies has been presented in the supplementary file Table S1. Over the years, solid-state approaches like mechanochemical alloying has been used to synthesize (Bi,Sb)_2_Te_3_, however, the risk of contamination, high cost of high-energy ball milling and longer hours of ball milling, and significant batch to batch variations make this method inconvenient [24,25]. Bottom up chemical methods are commonly used because of their low cost, low investment need and possibility of scale-up of the synthesis process [26]. Different morphologies of Bi_2−x_Sb_x_Te_3_ including nanoparticles, nanoplates, nanocrystalline films, nanorods, nanotubes, nanowires and nanoflowers have been synthesized by reverse micelle [27], metal-organic chemical vapor deposition (MOCVD) [28,29,30], vapor–liquid–solid (VLS) [31,32], refluxing [33,34,35,36], electrodeposition [37,38], chemical reduction [39,40], solvothermal [41,42,43,44,45,46,47,48,49], hydrothermal [26,50,51,52,53,54,55,56,57,58], ultrasonic-assisted [59,60,61], and microwave (MW)-assisted [62,63,64,65,66] routes, to name a few. Among all these chemical syntheses methods, solvothermal/hydrothermal routes are the preferred methods to prepare nanostructures with different morphologies. However, a major drawback in solvothermal/hydrothermal methods is the reaction time that can range from several hours to days [26,42,46,52,57,58]. This shortcoming can be resolved by introducing energy-effective MW-assisted heating.

MW technology is emerging as an alternative energy source for chemical synthesis with the aim to reduce the reaction time from hours or even days to a few minutes [64,65,67,68,69,70]. The dielectric constant of the solvent may significantly influence the outcome of the MW-assisted reaction. MW speeds up the chemical reaction by increasing the overall kinetics of the reaction as it relies on localized superheating [71]. Other advantages of using MW heating are higher yields, and since the reaction is kinetically driven it ensures a narrow-size distribution of the particles [72]. A variety of nanomaterials have been synthesized with MWs, including 0D [63,72,73,74,75,76,77,78], 1D [67,68,79,80] and 2D structures [81]. MW-assisted synthesis is also a sustainable, environmentally friendly, and economically viable heating method for bulk commercial scale production of strategic nanomaterials.

Most chemical syntheses use a large amount of conventional volatile or other organic chemicals, which create a pressing environmental concern. Therefore, the aim should be to minimize the use of toxic chemicals and follow the principles of green chemistry to synthesize nanomaterials [82]. The selection of the solvent for a reaction can dramatically affect the reaction outcome. Ethylene glycol (EG) is an abundant resource in the chemical industry, which can be produced from renewable biomass [83]. It is an odourless, non-volatile, low toxic solvent with a low dielectric constant (37 at room temperature), which has enormous applications in various industrial processes [84], including the polyol synthesis [64]. Water is the most environmentally friendly (i.e., green) solvent with a high dielectric constant (78 at room temperature), which is very effective for transferring electromagnetic energy into heat and thus driving the reduction reactions effectively [85]. Under MW heating, the superheating of water causes the hydrogen bonds to break easily, making it less polar and the self-ionization of water increases with temperature [62,86]. Due to superheating, the dielectric constant of water decreases at high temperatures (above its boiling point) and this influences the growth of the nanoparticles [86]. It has been reported that Bi_2_Te_3_ particles grow more steadily in a solvent with low dielectric constant than in a solvent with a high dielectric constant [41]. Therefore, this makes the combination of MW heating and hydrothermal methods a suitable chemical synthesis route for the growth of nanocrystals. The pH of the reaction medium is key for the synthesis of the Bi_2_Te_3_ nanocrystals. It has been reported that an alkaline medium (pH < 11–12) is required for the formation of Bi_2_Te_3_ nanostructures [26,43,52,53,57,87]. The alkaline medium helps the tellurium to disperse in the reaction medium as telluride ions (Te^2−^), which is beneficial for the formation of the nanocrystals [46]. A growth promoting agent, like ethylenediaminetetraacetic acid (EDTA), is required to form the bismuth-antimony complexes which self-assemble to lamellar phases leading to the formation of Bi_2_Te_3_ sheet nuclei, which facilitates the formation of nanosheets, nanoflakes and nanotubes [53,59]. The activity of EDTA is also dependent on the pH of the solution and by changing the pH, different morphologies of Bi_2_Te_3_ have been obtained [51,52,53,54,55,56,87]. Control of the reaction time and temperature are essential in determining the structure and morphology of the nanocrystals [49,57]. It can be deduced that low temperature is suitable for the reaction of nanoparticles, nanoplates and nanotubes and as the reaction temperature increases, the dimensionality changes from 0D to 1D. The effect of reaction time and temperature on the morphology, structure, and crystallinity of Bi_2_Te_3_ have been comprehensively summarized in Table S1.

We aim to provide facile and sustainable synthetic routes for large-scale production of Bi_2_Te_3_ nanomaterials with promising TE performance. The design of the experiments has been done in-line with the green chemistry perspectives considering the solvents, precursors and the energy source. Taking advantage of the unique MW dielectric heating phenomenon, rapid heating and short reaction time, hexagonal nanoplatelets of Bi_2_Te_3_ were obtained. These low dimensional structures have been effective in enhancing the charge transport properties and electrical conductivity, while reducing the thermal conductivity. Water and ethylene glycol are used as solvents making the reaction green, safe and non-hazardous, producing high purity TE nanopowders in a matter of minutes due to very effective volume heating—despite their significantly different dielectric constants. Besides yielding a large quantity of the investigated materials per batch, surface chemistry and the processing of these materials towards compacts are described, resulting in high ZT values of 1.03 and 0.88 for Bi_2_Te_3_ materials synthesized through the polyol and the hydrothermal routes, respectively.

## 2. Materials and Methods

### 2.1. Synthesis and Processing of Thermoelectric Materials

#### 2.1.1. Synthesis by Hydrothermal Method

**Figure 1:**
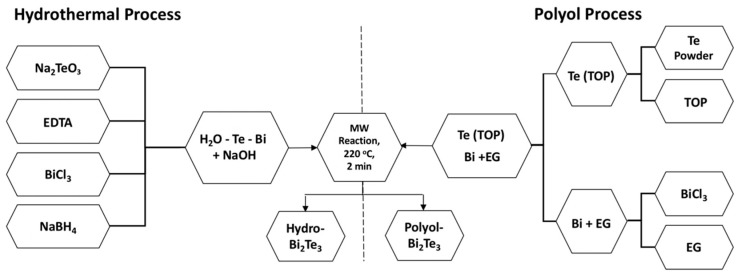
Schematic of the MW-assisted hydrothermal, and polyol synthesis process.

Chemicals used for the synthesis were of analytical grade obtained from Sigma Aldrich (Stockholm, Sweden) and have been used as received without further purification: Bismuth chloride (BiCl_3_, 98% purity), sodium tellurite, (Na₂TeO₃, 99% purity), sodium hydroxide (NaOH, 97.0% purity) and sodium borohydride (NaBH_4_, 98% purity). BiCl_3_ and Na₂TeO₃ were used as the precursors for Bi and Te ions, respectively. Deionized (DI) water was used as the solvent, EDTA as the structure-directing agent, NaBH_4_ as the reducing agent and NaOH to control the pH of the reaction media. The synthesis was performed in a Milestone flexiWAVE MW system (MILESTONE Srl, Sorisole, Italy) (900 watt magnetrons for a total of 1800 watt). One-pot synthesis was achieved by mixing all the precursors in the same media. For the synthesis of Bi_2_Te_3_, Bi:Te molar ratio of 2:3 was used. In a 100 mL Teflon vial, BiCl_3_, Na₂TeO₃, EDTA, and NaBH_4_ were mixed where the pH was adjusted to 10.5–11 by the addition of NaOH. Two parallel reaction vessels were used in a single run. The reaction vials were kept under constant magnetic stirring for 30 min to ensure a homogeneous mixing. This was then subjected to MW heating to 220 °C with a ramp time of 4 min and dwell time of 2 min. The solution was then allowed to cool down to room temperature and a clear phase separation was observed with the product settled at the bottom, separated from water. The particles formed were easily separated from the reaction mixture by centrifugation and were then washed with isopropanol, acetone and water. Thereafter, the powder was dried in a vacuum oven at 60 °C. A schematic of the hydrothermal synthesis process is given in Figure 1. The sample is designated as hydro-Bi_2_Te_3_ throughout the manuscript.

#### 2.1.2. Synthesis by Polyol Method

All the chemicals used for the synthesis reaction were of analytical grade obtained from Sigma Aldrich and were used as received without further purification: BiCl_3_ (98% purity), Te powder (99.8% purity), ethylene glycol (EG, 99% purity thioglycolic acid (TGA, 98% purity), and trioctyl phosphine (TOP, 90% purity). The synthesis was performed in the same Milestone flexiWAVE MW system, as described in our earlier work [64]. Typically, BiCl_3_ was dissolved in EG as a source of Bi^3+^ ions, by high power sonication for 2 min, then TGA was added under continuous stirring. Te precursor solution was prepared by complexing the Te powder with TOP using MW assisted heating for 90 s at 220 °C. The molar ratio of 2:3 was used for the concentration of Bi:Te respectively. Both precursors were mixed in a 100 mL Teflon vial, then loaded into the MW system heated to 220 °C with a ramp time of 4 min and dwell time of 2 min. Thereafter, the powders were washed several times with acetone and isopropanol and dried in a vacuum oven at 60 °C. A schematic of the polyol synthesis procedure is given in Figure 1. The sample is designated as polyol-Bi_2_Te_3_ throughout the manuscript.

### 2.2. Consolidation of the Powders via Spark Plasma Sintering

The powders were then compacted by spark plasma sintering (SPS, Dr Sinter 825, Fuji Electronic Industrial Co. Ltd., Tokyo, Japan), to obtain nanostructured bulk solid materials, in a 15 mm diameter die to form pellets for TE transport property measurements (see Figure S1). Bi_2_Te_3_ powders from both the hydrothermal and the polyol syntheses methods were subjected to the same SPS process parameters for a fair comparison (see Table 1). The sintering temperature was set to 400 °C with the ramping heat rate of 30 °C/min, and 50 MPa pressure applied with the same ramping rate, as to reach the maximum pressure when the temperature reached a maximum 400 °C. The sintered pellets were cooled down to room temperature and polished for further structural analysis and TE transport measurements. The density (d_pellet_) of the sintered specimens was measured with the Archimedes method and the packing density (ρ%) was calculated with reference to the bulk density (d_bulk_) of 7.86 g/cm^3^.

### 2.3. Structural and Morphological Characterization

The phase structures are investigated by X-ray powder diffraction (XRPD) to identify the crystal structure, crystallinity, and the lattice parameters and compare the purity of the synthesized materials. XRPD analysis has been performed by using a Philips PANalytical X’Pert Pro Powder Diffractometer (Malvern Panalytical Ltd., Malvern, UK) with Cu-Kα_1_ radiation (λ = 1.54059 Å). A scan speed of 0.04°/s was used in continuous scan with a rotating sample holder. Morphology and microstructure analyses of the materials were performed using scanning electron microscopy (SEM; FEI Nova 200 (FEI Company, Hillsboro, OR, USA) and FE-SEM, TESCANBRNO-Mira3 LMU (TESCAN ORSAY HOLDING, Brno, Czech Republic) equipped with a 30 kV SEM FEG column. During the operation of the instrument, the working distance was maintained at 5 mm and the accelerating voltage was 10–15 kV. Transmission electron microscopy (TEM, JEM-2100F, JEOL Ltd., Tokyo, Japan) analysis was performed by pipetting a 100 μL aliquot of the powder samples dispersed in DI water onto a 200 mesh Cu-grid and drying prior to analysis.

Zeta (ξ) potential of various samples were measured at room temperature in triplicates with Malvern Zetasizer Nano ZS90 at an incident angle of 90°. All samples were dispersed in DI H_2_O, and titrated with 0.01 M HCl or 0.01 M NaOH to obtain pH dependent surface charge, and eventually to determine the isoelectric point (iep). The term iep represents a specific pH value where the net surface charge of the particle is zero, which is strongly linked to the surface chemistry and functionality on the nanoparticles.

### 2.4. Electronic and Thermal Transport Property Measurements

Electrical conductivity (σ) and Seebeck coefficient (S) measurements were performed simultaneously by using the commercial ZEM3 ULVAC-RIKO (ULVAC, Methuen, MA, USA) system. The measurements were conducted on SPS consolidated pellets in the system for both (hydrothermal and polyol) samples. The total thermal conductivity κ_tot_ of the pellets were calculated according to the equation κ_tot_ = C_p_·α·ρ, where C_p_, α and ρ are specific heat capacity, thermal diffusivity, and density of mass respectively. Heat capacity measurements were performed with differential scanning calorimetry (DSC, PT1000 Linseis, Linseis Messgeraete GmbH, Selb, Germany). For measurement of the thermal diffusivity, α, 2 mm thick disk-shaped samples were used in the laser flash analysis (LFA 1000, Linseis) system.

### 2.5. X-ray Photoelectron Spectroscopy

X-ray photoelectron spectroscopy (XPS) was used for surface analysis. The XPS spectra were acquired using the PREVAC 426 system configuration, equipped with SCIENTA R3000 hemispherical photoelectron spectrometer (Scienta Omicron, Uppsala, Sweden) and monochromatic Al source. The base pressure during measurements was better than 3 × 10^−10^ mbar. All acquired spectra were calibrated to adventitious carbon C1s at 285 eV. After subtraction of the Shirley-type background, the core-level spectra were decomposed into main components with mixed Gaussian–Lorentzian lines (70% G + 30% L for majority of photo-peaks) by a non-linear least squares curve-fitting procedure, using CasaXPS software (version 2.3.18, Casa Software Ltd., Cheshire, UK).

## 3. Results and Discussion

### 3.1. Structural Analysis

**Figure 2:**
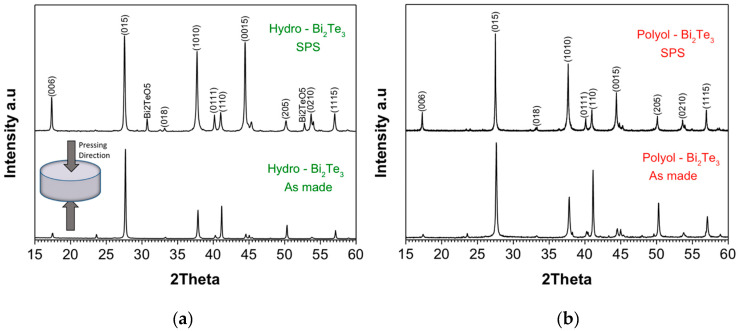
X-ray powder diffraction patterns (XRPD) (normalized for the intensity of the most intense 015 peak) of as-synthesized and spark plasma sintered Bi_2_Te_3_ samples synthesized through MW-assisted heating for (**a**) hydro-Bi_2_Te_3_, and (**b**) polyol-Bi_2_Te_3_ sample. The major crystalline phases are indexed to Bi_2_Te_3_ (ICDD: 01-089-2009) with rhombohedral crystal structure (a = b = 4.3860 Å, c = 30.4970 Å; α = β = 90°, γ = 120°).

Structural analysis of as-synthesized materials through MW-assisted hydrothermal and polyol routes before and after SPS sintering are performed using XRPD. The diffraction patterns are presented in Figure 2, where the patterns are normalized for the most intense peak with Miller indices (015). As for the SPS sintered samples, XRPD was performed on the pellets’ surface perpendicular to the sintering direction (pressing axis/direction is schematically shown in the inset of Figure 2a). The crystalline phases are indexed to Bi_2_Te_3_ (ICDD: 01-089-2009) with rhombohedral crystal structure, and the corresponding indexing of the Bragg diffractions with relevant Miller indices are shown on the diffraction patterns in Figure 2. When as-made samples are compared, it can be clearly seen that polyol-Bi_2_Te_3_ sample has more intense peaks for the Bragg diffractions with the same Miller indices (e.g., (1010), (110), (205)), which reveal a higher crystallinity as compared to the hydro-Bi_2_Te_3_ sample. Upon sintering, the significant increase in the relative intensity of diffraction peaks with (001) index reveal a higher degree of texturing in the hydro-Bi_2_Te_3_ sample. The intensity ratio of (006) peak to (015) peak could be used for the quantification of the texturing. The hydro-Bi_2_Te_3_ sample has the peak intensity ratio (I_006/_I_015_) of 0.38, while it is 0.19 for the polyol-Bi_2_Te_3_ sample (see Figure S2). This indicates that after the SPS process the c-axis of the grains was preferentially oriented parallel to the pressing direction, resulting in a higher degree of texturing in the Hydro-Bi_2_Te_3_. Average crystallite size of the sintered samples was estimated from the XRD data using the Williamson–Hall model [88], as 231 nm for the polyol-Bi_2_Te_3_ sample, and 295 nm for the hydro-Bi_2_Te_3_ sample. A minor/secondary phase of Bi_2_TeO_5_ observed in the hydro-Bi_2_Te_3_ sample upon sintering. This phase was also observed in some earlier reports [89,90,91], and is estimated as 5.8% of the overall composition through the Rietveld refinement (see Figure S3). Possible reasons to observe Bi_2_TeO_5_ (ICDD: 00-024-0154) impurity phase after the SPS process may be either due to crystallization of the pre-existing secondary phase upon sintering, or to the reaction of the powder with the trace amount of oxygen in the sintering medium.

### 3.2. Surface Analysis

**Figure 3:**
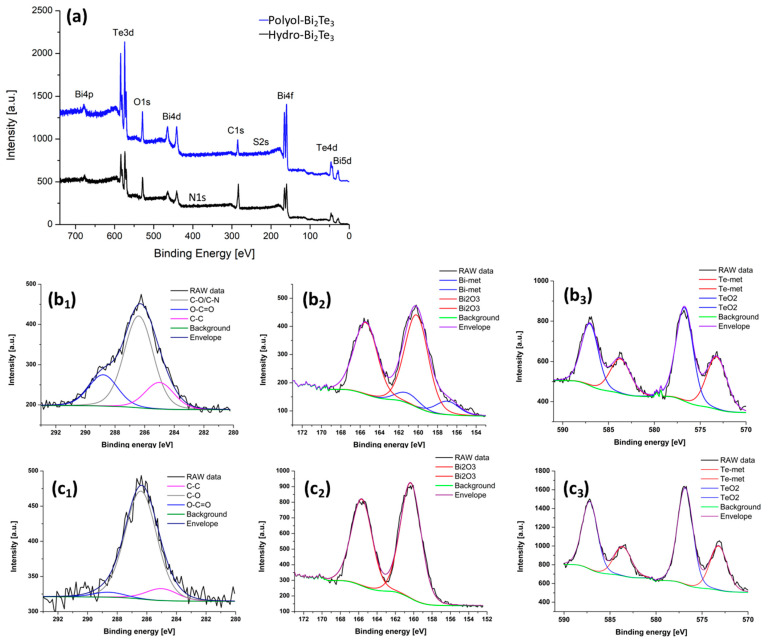
X-ray photoelectron spectroscopy (XPS) survey scan (**a**), and C 1s (**b_1_**,**c_1_**), Bi 4f (**b_2_**,**c_2_**) and Te 3d (**b_3_**,**c_3_**) spectra for Bi_2_Te_3_ samples synthesized through MW-assisted hydrothermal (**b**), and polyol (**c**) routes.

XPS analysis was performed on as made powder samples synthesized through hydrothermal and polyol routes. The data are graphically presented in Figure 3 and are summarized in Table 2. It should be noted that in both the samples tellurium (Te) exists in two states, metallic and oxide. In the case of bismuth (Bi) content, the hydro-Bi_2_Te_3_ sample consists of Bi in two states, metallic and oxide, while in the case of the polyol-Bi_2_Te_3_ sample the element Bi exists only in the oxide state. The speciation of carbon (C) is slightly different in the materials, where it is a mixture of mainly C–O, and C–C for polyol-Bi_2_Te_3_, while it consists of C–O, C–C and higher O–C=O content for the hydro-Bi_2_Te_3_ sample. The chemical speciation of C on the surface can be seen from the XPS fitting details, where a high percentage of C–O was identified in the hydro-Bi_2_Te_3_ sample, followed by C=O groups. This may be due to a strong conjugation of the terminal carboxyl groups to the nanoparticles’ surface through a bidentate or bridging configuration. In the polyol sample though, C is dominantly indexed to carboxyl, O–C=O, which is reasonable considering the conjugation of thioglycolic acid to the NP surface being through the sulfur (S) atoms. C content in the hydro-Bi_2_Te_3_ sample is higher than that of the polyol sample. This may originate from the precursors used in the synthetic process. Indeed, trace elements originating from the precursors are detected in the spectra of the respective samples. EDTA was used as a surface directing agent in the hydrothermal synthesis, which is known to be a hexadentate ligand at pH above 10. Naturally, it stays on the formed hexagonal platelets as an integral part, despite several washing-re-dispersing steps. The traces of EDTA can be seen through the nitrogen (N) signal (400.5 eV) in the XPS spectrum of the hydro-Bi_2_Te_3_ sample (Figure 3a). In the polyol process, thioglycolic acid was used as a directing agent and its traces are also visible through the sulfur (S) signal (229.3 eV) in the XPS spectrum of this particular sample (Figure 3a).

It is important to note that the relative ratio of metallic vs. oxide phases on the NPs’ surface reveals that the polyol-Bi_2_Te_3_ sample is bearing more oxide phase than the hydro-Bi_2_Te_3_ sample. In the case of Bi in particular, the spectrum for polyol-Bi_2_Te_3_ (Figure 3c_2_) indicates Bi_2_O_3_ as the only identified phase. It is interesting to note this as water has been generally considered a stronger oxidizing solvent.

**Figure 4:**
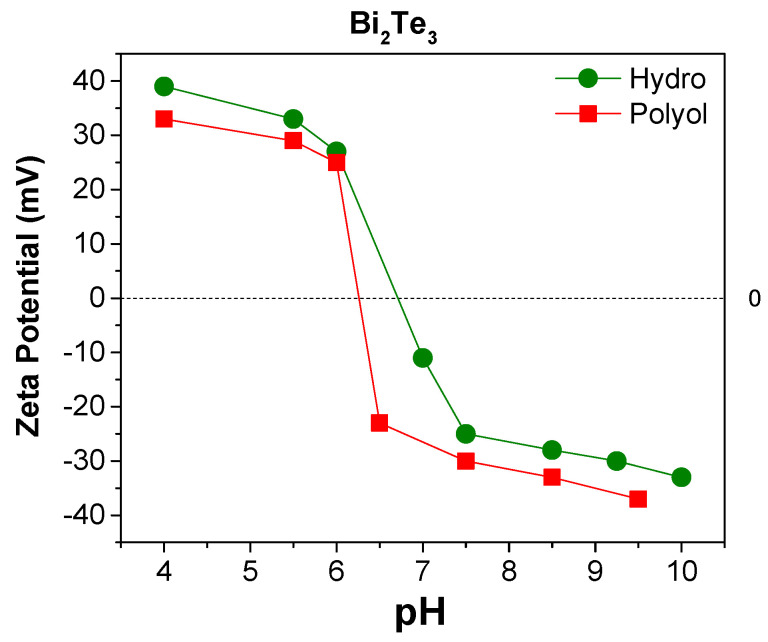
ξ-potential analysis of as-made Bi_2_Te_3_ samples synthesized through MW-assisted hydrothermal, and polyol routes.

Zeta (ξ) potential analysis was performed on both hydrothermal and polyol as-made powders dispersed in DI water. Isoelectric point (iep) is strongly dependent on the surface chemistry of the particles, and the dispersant. Determining the iep of as-synthesized Bi_2_Te_3_ materials can reveal the information about the surface charge and chemistry of the as-made nanoparticles. For the polyol synthesized sample, the iep was reached around pH 6.3, while it was about 6.8 for the hydrothermally synthesized sample (Figure 4). These values are not significantly different, and both may derive from the high C content, carbonyl and carboxyl bearing surface groups. The small difference can be attributed to the TeO_2_, which has an iep of <3. The higher TeO_2_ content of polyol-Bi_2_Te_3_ sample can explain the slightly lower iep value.

### 3.3. Microstructure Analysis

**Figure 5:**
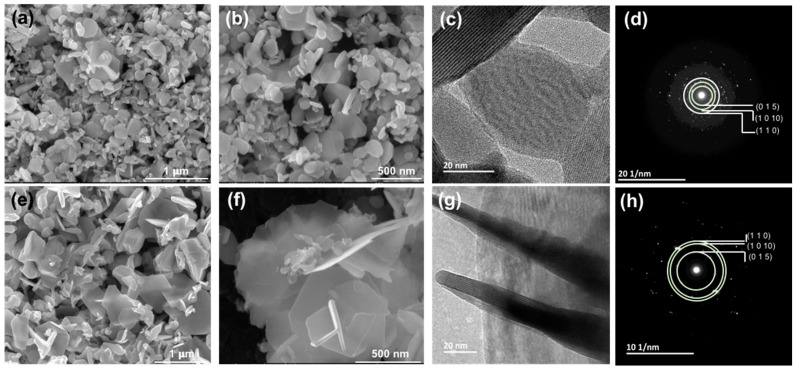
Scanning electron microscopy (SEM) micrographs of as-made Bi_2_Te_3_ samples at different magnifications; (**a**,**b**) hydro-Bi_2_Te_3_ and (**e**,**f**) polyol-Bi_2_Te_3_. (**c**,**g**) Transmission electron microscopy (TEM) micrographs and (**d**,**h**) selected-area electron diffraction (SAED) patterns of hydro-Bi_2_Te_3_ and polyol-Bi_2_Te_3_ samples, respectively.

Morphology and microstructure analyses of as-made nanoparticles and sintered samples were performed using SEM and few micrographs for powder samples at different magnifications for hydro-Bi_2_Te_3_ and polyol-Bi_2_Te_3_ samples are given in Figure 5. Both the samples show dominantly hexagonal platelet morphology for the nanoparticles formed. The Bi_2_Te_3_ crystals grow predominantly along the ab plane direction because the strong covalent bonds between Bi–Te can be easily extended along the plane. The platelets obtained in the polyol route were observed to be thinner than those from the hydrothermal route. In general, particles obtained from the hydrothermal route are smaller than those from the polyol route. The lateral dimensions show a great variation in the range 20–250 nm for the hydrothermal and 10–1000 nm for the polyol sample, respectively. These variations are attributed to the different solvents and the stabilizing ligands used in the two syntheses process, which have had a different impact on the nucleation and growth kinetics. Looking closer to the XRPD patterns in Figure 2, it can be assessed that the diffraction peak at 41°, corresponding to the 110 Bragg diffraction, is much higher for the polyol sample, ascribed to larger lateral dimension (ab plane) of the platelets. A closer investigation was performed by TEM analysis and micrographs along with selected-area electron diffraction (SAED) patterns are shown in Figure 5c,d,g,h for hydro-Bi_2_Te_3_ and polyol-Bi_2_Te_3_ samples, respectively. Despite their large lateral dimensions, the platelets exhibit almost perfectly single crystalline character. There are some platelets aligned sideways, which reveal clearly the lamella-like structure along the c-axis, with a typical thickness of about 1 nm, as shown in Figure 5c,g. These layers are five atomic layers (Te-Bi-Te-Bi-Te) thick, which are known as the quintuple layer, held together by the van der Waals forces. SAED patterns, presented in Figure 5d,h, are indexed to typical Bragg diffractions of rhombohedral Bi_2_Te_3_ (ICDD: 01-089-2009) as was also identified in the XRPD patterns (Figure 2).

**Figure 6:**
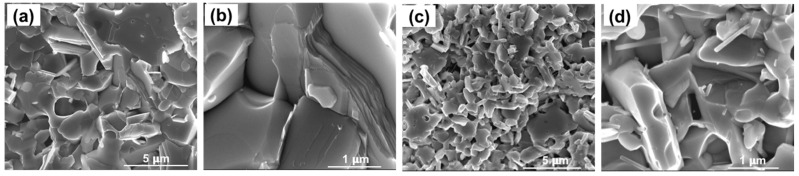
SEM micrographs of SPS sintered Bi_2_Te_3_ pellets at different magnifications for materials synthesized through MW-assisted hydrothermal (**a**,**b**) and polyol (**c**,**d**) routes.

The as-synthesized nanostructured TE materials were consolidated using the SPS. The critical consolidation parameters (sintering temperature, holding time, pressure, and heating rates), and characteristics of the resultant pellets are presented in detail in Table 1. Despite both the samples being processed under identical conditions, the pellets reveal a big difference in their packing density; a packing density of about 80% for the polyol-Bi_2_Te_3_ and 90% for the hydro-Bi_2_Te_3_ sample. Densification under SPS for a given material as a function of particle size has been studied by Diouf et al. [93], where they reported a decrease in densification with increasing particle size of the specimen. In the case of the Bi_2_Te_3_ samples, particle size of the polyol-Bi_2_Te_3_ sample was assessed to be larger than the hydro-Bi_2_Te_3_ sample, which may be the cause of the observed difference in the densification, in agreement with the earlier findings [93]. Microstructure of the pellets are displayed in Figure 6. The hydro-Bi_2_Te_3_ sample shows a clear long-range stacking of hexagonal platelets, perpendicular to the c-axis, revealing a higher degree of texturing as compared to the polyol-Bi_2_Te_3_ sample. This effect was observed in XRD (Figure 2), with a significant increase in the intensity ratio (I_006_/I_015_) of the diffraction peaks for the hydro-Bi_2_Te_3_ sample (see Figure S2).

### 3.4. Electronic Transport Property Evaluation

**Figure 7:**
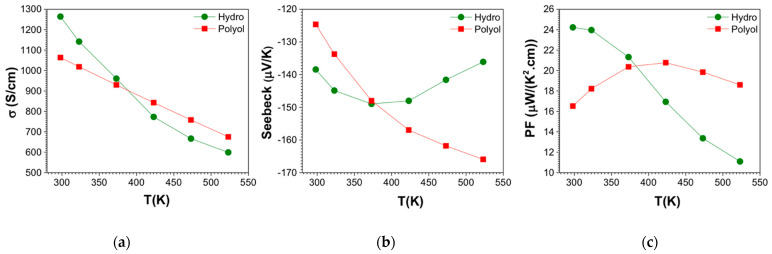
Temperature-dependent electronic transport properties of SPS sintered Bi_2_Te_3_ pellets for Bi_2_Te_3_ materials synthesized through MW-assisted hydrothermal and polyol routes; (**a**) electronic conductivity, (**b**) Seebeck coefficient, and (**c**) power factor.

The electronic transport data in the temperature range of 300–523 K are presented in Figure 7. Table 3 summarizes the transport data at room temperature and at the temperature where the maximum ZT was obtained. No adverse effects in the transport properties were observed due to the presence of surface oxides in the as-made powders. The electrical conductivity (σ) is plotted in Figure 7a, where samples show reduction of σ by increasing temperature, which is typical of heavily doped semiconductor, or metallic character. The highest electrical conductivity value at room temperature was measured for hydro-Bi_2_Te_3_. The σ for hydro-Bi_2_Te_3_ changes from about 1300 S/cm at 300 K to about 600 S/cm at 523 K. This is likely due to the higher density of the compacted materials and larger layered structure of the hydro-Bi_2_Te_3_ in comparison to the polyol-Bi_2_Te_3_ sample. The secondary phase of Bi_2_TeO_5_ in the hydro-Bi_2_Te_3_ sample is an oxide with a larger band-gap than Bi_2_Te_3_ [90,94]. Due to its low content within the main phase, it is not expected to have a percolation path to influence the electronic transport properties significantly. This can also be seen from the initial high σ of hydro-Bi_2_Te_3_. However, this oxide phase may act as phonon scattering canters due to its different anisotropic crystal structure, influencing the thermal conductivity. The σ values change between 1100 S/cm and 700 S/cm for the polyol-Bi_2_Te_3_ sample, in the same temperature range. Reduction in the electrical conductivity over 300 K is generally a signature of increasing electron-phonon scattering. There is an interesting crossover at 373 K, where the σ for the hydro-Bi_2_Te_3_ sample goes below the polyol sample. This might be due to the electron-phonon scattering process being higher than the polyol-Bi_2_Te_3_ sample. The electrical conductivity of both samples are higher than those of other solution derived samples [95,96,97] comparable with that of p-type alloy ingot (on the level of 1 × 10^3^ S/cm at 300 K) [98].

The Seebeck coefficient (*S*) is the key parameter to demonstrate the type of transport. Figure 7b shows the obtained *S* values for both samples, which have negative values, revealing n-type conduction. The polyol-Bi_2_Te_3_ sample starts the *S* from −120 μV/K at room temperature and increases in magnitude up to −165 μV/K at 525 K, while the hydro-Bi_2_Te_3_ sample does not show a strong temperature dependence, displaying values between −140 μV/K and −150 μV/K. Electronic transport properties are strongly correlated with the microstructure, as well as charge carrier density, defects, and scattering events. This correlation is clearly visible for the hydro-Bi_2_Te_3_ sample, where a decreasing σ is accompanied by an increase in the magnitude of the *S*. The band-gap (E_g_) can be estimated from the *S* as it is sensitive to changes in the density of states near the Fermi level [99,100]. The E_g_ for the materials were calculated using the electron charge (q), absolute value of the maximum Seebeck, |*S_max_*|, value and the corresponding temperature $T_{max}$, using the following equation:

(1) $${\;E}_{g}=2q|S_{max}|\cdot T_{max}$$

The estimated E_g_ from the S*_max_* for the hydro-Bi_2_Te_3_ sample is 0.11 eV and that for the polyol-Bi_2_Te_3_ sample is 0.17 eV. The estimated E_g_ for the Hydro-Bi_2_Te_3_ sample is in good agreement with the calculated E_g_ of 0.11 eV by Mishra et al., using the density-functional theory with the spin–orbit interaction included [101]. However, the E_g_ for the hydro-Bi_2_Te_3_ sample is lower than the bulk Bi_2_Te_3_ value of E_g_ = 0.15 eV [102], while that of the polyol-Bi_2_Te_3_ sample is slightly higher. The nanocrystal size effect on the shifts of valence and conduction band edge and the band-gap expansion of semiconductor nanocrystals has been modeled by Lang et al. [103]. This model, without any adjustable parameter, predicted that the band-gap expansion (along with shifts of valence and conduction band edges) is present with dropping size of nanocrystals because of the increase of surface/volume ratio. Crystallite size of the sintered pellets has been estimated as 295 nm for the hydro-Bi_2_Te_3_ sample, and 231 nm for the polyol-Bi_2_Te_3_ sample. Therefore, the estimated E_g_ values agree with the trend expected from the relative size of the crystallites in the studied materials.

The power factor (PF) (*S*^2^ σ) calculated for both hydrothermal and polyol samples, as presented in Figure 7c. The highest PF at room temperature was obtained for the hydro-Bi_2_Te_3_ sample as 24 μW cm^−1^ K^−2^, followed by the polyol-Bi_2_Te_3_ sample as 16 μW cm^−1^ K^−2^. The maximum power factors we achieved are 24 μW cm^−1^ K^−2^ for the hydrothermal sample at 300 K and 21 μW cm^−1^ K^−2^ at 423 K for the polyol sample. The magnitude of PF is comparable to or much higher than other solution-derived samples—with maximum power factor 1–9 μW cm^−1^ K^−2^ [66,96].

### 3.5. Thermal Conductivity

**Figure 8:**
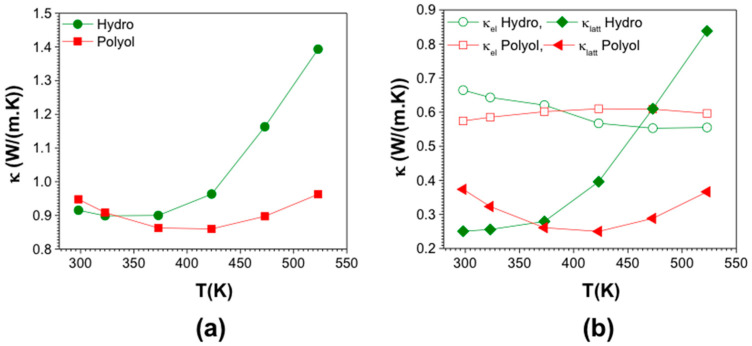
Total thermal conductivity (**a**), along with the electronic and thermal contributions to the thermal conductivity (**b**) for SPS compacted pellets for hydrothermal and polyol synthesized Bi_2_Te_3_.

The total thermal conductivity values were calculated using the thermal diffusivity, heat capacity and mass density values of the samples. The total thermal conductivity of hydro-Bi_2_Te_3_ is slightly lower than polyol-Bi_2_Te_3_ at T = 293 K, as seen in Figure 8a. They both decrease first and then increase with increasing temperature, where it shows a dramatic increase for the hydro-Bi_2_Te_3_ sample after 373 K. Nanostructures at different length scales (diameter, roughness and point defects) showed efficient scattering throughout the phonon spectrum, reaching very low thermal conductivity values [23]. Looking at the microstructure of the pellets obtained, there could be a mixture of these nanostructures at different length scales, influencing the phonon propagation. The actual nanostructures include undefined structural features, such as interface defects, impurities, strain, etc., making it difficult to classify the contribution of each scattering process to phonon transport [104]. In order to make a clear assessment, we have examined lattice (κ_lat_) and electronic thermal conductivity (κ_el_) of both the samples by using the Wiedemann–Franz relationship, κ_el_ = *L*_o_*σ*T where *L*_o_, *σ*, and T are Lorenz number, the electrical conductivity and the absolute temperature, respectively. The κ_lat_ values were estimated by subtracting the κ_el_ from the total thermal conductivity. In order to calculate the Lorenz number (*L*_o_), experimentally obtained Seebeck coefficient values were used and a single parabolic band model was assumed to solve the Fermi integral. Lorenz numbers for both the samples are graphically presented in Figure S4. The κ_lat_ and κ_e_ are shown in Figure 8b. Since the κ_el_ is determined by the σ, it is expected to possess a similar trend to the electrical conductivity. In both the samples the κ_el_ contribution is higher than the lattice contribution, κ_lat_, most likely due to the electrons’ more dominant role on the thermal conductivity. The striking feature is the very low lattice contribution in these samples, strongly influenced by the increased density of grain boundaries that plays a crucial role in increased phonon scattering and thus a strong reduction in κ_lat_. The room temperature κ_latt_ for the hydro-Bi_2_Te_3_ and polyol-Bi_2_Te_3_ were about 0.25 W/m·K and 0.37 W/m·K, respectively. The κ_lat_ of the hydro-Bi_2_Te_3_ sample increases with temperature after 373 K. This might be due to the highly crystalline texture (long layered structure) thus longer MFP of phonons. Crystallite size of the sintered pellets has been estimated as 295 nm for the hydro-Bi_2_Te_3_ sample, and 231 nm for the polyol-Bi_2_Te_3_ sample. The difference in the E_g_ is the evidence of the correlation of the E_g_ with the average crystallite size; a smaller crystallite size leading to a larger band-gap and vice versa. Due to the smaller E_g_ of the hydro-Bi_2_Te_3_ sample (0.11 eV), at elevated temperatures the minority carriers’ diffusion (ambipolar effect characteristic for the electron-hole pair diffusion on the onset of the intrinsic conduction) contributes strongly to the total thermal conductivity κ_tot_ [105,106].

Regardless of the synthetic route, the total thermal conductivity of the materials is lower than that of the current best-known n-type Bi_2_Te_2.925_Se_0.075_ (κ_tot_ = 1.65 W/m·K at 300 K in which the κ_lat_ was 1.27 W/m·K) [16], and are also comparable to those of ball milled and dc hot pressed n-type Bi_2_Te_2.7_Se_0.3_ bulk samples (κ_tot_ = 1.06 W/m·K at 300 K in which κ_lat_ was 0.7 W/m·K) [107]. This strongly suggests that the nanostructure obtained through the developed synthetic routes plays a very significant role in reducing the κ_lat_ through effective phonon scattering.

**Figure 9:**
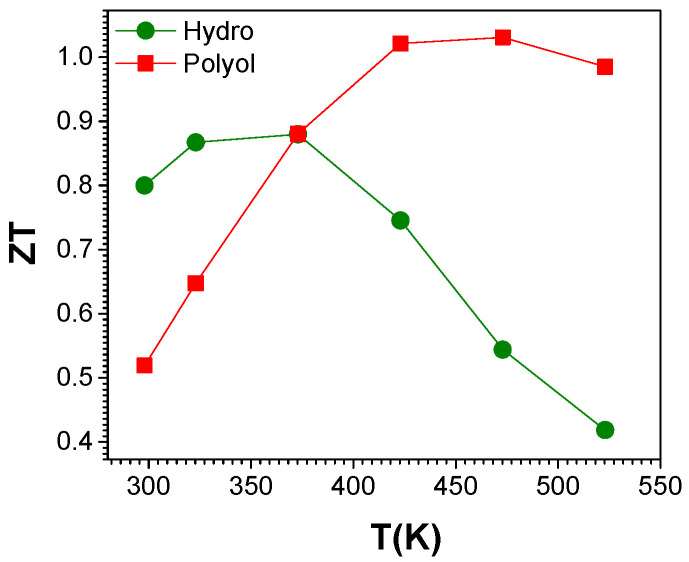
Temperature dependent thermoelectric (TE) figure of merit (ZT), of SPS compacted pellets for Bi_2_Te_3_ materials synthesized through MW-assisted hydrothermal and polyol routes.

Temperature dependence of the estimated ZT values for the samples are shown in Figure 9. Room temperature ZT values of 0.8 and 0.52 were obtained for hydro-Bi_2_Te_3_ and polyol-Bi_2_Te_3_ samples, respectively. The maximum ZT values were obtained at slightly different temperatures, in the range 323–373 K for hydro-Bi_2_Te_3_, and 423–473 K for polyol-Bi_2_Te_3_. This is mainly because the samples display a maximum power factor and a minimum thermal conductivity at different temperatures. The maximum ZT value reaches about 0.9 at 373 K for hydro-Bi_2_Te_3_ while for polyol-Bi_2_Te_3_ it is about 1.03 at 473 K. These are among the highest ZT values reported for n-type Bi_2_Te_3_ in the literature. This study shows two viable synthetic strategies, which yield materials with promising efficiency at slightly shifted temperatures that can be used for guiding the synthetic route for the intended temperature range of applications.

## 4. Conclusions

Rapid and scalable synthetic strategies for high-quality and reproducible thermoelectric (TE) materials are demonstrated in this work, exemplified by nanostructured n-type bismuth telluride with a promising TE performance. Use of microwaves enables an effective volume heating, leading to highly crystalline TE nanopowders in a few minutes. Polyol synthesis is shown to be a reliable and scalable route to materials synthesis. Hereby we show that not only EG, but also water is an excellent reaction media, which is certainly much more benign and abundant than any other solvent. Furthermore, hydrothermal synthesis is one-pot process, where all the precursors are mixed in the same reaction medium prior to heating, which makes this process facile and therefore more attractive. Despite the significant difference in their dielectric constant, both solvents (EG and water) showed impressive outcomes for the materials investigated. Crystal structure, crystallinity, morphology, microstructure and surface chemistry of the synthesized materials were evaluated using a library of analytical techniques, including XRD, SEM/TEM, XPS and zeta potential techniques. Particles with dominantly hexagonal platelet morphology were obtained, where the polyol-Bi_2_Te_3_ generally exhibited a larger particle size than the hydro-Bi_2_Te_3_. Surface chemistry studied by XPS clearly revealed different levels of surface oxidation, based on the solvent used, and traces of the precursors used on the surface of as-synthesized materials. Surface directing agents are stably bound to the surface of the as-formed nanoparticles, thus assuring the maintenance of the hexagonal platelet morphology. The samples were then consolidated using SPS, prior to further transport measurements. The difference in the particle size of the specimens significantly influenced the densification behavior under SPS, where polyol-Bi_2_Te_3_ with larger particle size reached about 10% lower densification compared to the hydro-Bi_2_Te_3_ sample under identical conditions. Upon sintering, texturing is observed to be more predominant in the hydro-Bi_2_Te_3_ sample, as assessed from XRD and SEM analyses. Thermoelectric transport properties were evaluated using electrical conductivity and Seebeck coefficient measurements up to 525 K, and the thermal diffusivity was assessed by using the LFA system. The final estimated TE figure-of-merit values about 0.8–0.9 was reached in the temperature range of 300–375 K for the hydro-Bi_2_Te_3_ sample, while 0.9–1 was reached in the temperature range of 425–525 K for the polyol-Bi_2_Te_3_ sample. Energy and time efficiency, scalability as well as the environmental impact of the synthetic processes presented here, along with the high ZT values achieved, pave the way for wider impact of these synthetic strategies for developing promising strategic materials in the field of thermal energy harvesting.

## Figures and Tables

**Table 1:** Spark plasma sintering (SPS) parameters for Bi_2_Te_3_ samples synthesized through microwave (MW)-assisted hydrothermal (hydro-Bi_2_Te_3_) and polyol (polyol-Bi_2_Te_3_) routes: sintering temperature (T_sint_), holding time (t_hold_), sintering pressure (P_sint_), bulk density (d_bulk_), density of pellets by the Archimedes method (d_pellet_), and packing density (ρ) in percentage of theoretical density.

	T_sint_	t_hold_	P_sint_	d_bulk_	d_Pellet_	ρ (%)
	(°C)	(min)	(MPa)	(g/cm^3^)	(g/cm^3^)	
Hydro-Bi_2_Te_3_	400	1	50	7.86	7.051	89
Polyol-Bi_2_Te_3_	400	1	50	7.86	6.13	78

**Table 2:** XPS peak fitting results for Bi 4f, and Te 3d, O 1s, C 1s, S 2s and N 1s regions, and the measured isoelectric point (iep) values for the Bi_2_Te_3_ samples synthesized through MW-assisted heating.

Samples	Fraction [at%]	Assigned to [92]		iep (pH)
Polyol-Bi_2_Te_3_	39.6	C	C–O, C–C,	6.30
			O–C=O	
	10.48	Bi	Bi_2_O_3_	
	25.86	Te	Te met (8.66%), TeO_2_ (17.2%)	
	22.29	O		
	1.77	S		
Hydro-Bi_2_Te_3_	61.53	C	C–O/C–N, C–C, O–C=O	6.80
	5.43	Bi	Bi met (0.6%), Bi_2_O_3_ (4.8%)	
	13.21	Te	Te met (5.3%), TeO_2_ (7.91%)	
	16.24	O		
	3.59	N		

**Table 3:** A summary of the measured TE transport properties of Bi_2_Te_3_ samples synthesized through MW-assisted hydrothermal and polyol routes: total thermal conductivity (κ_tot_), Seebeck coefficient (S), electrical conductivity (σ), power factor (PF), and TE figure of merit (ZT) at the given temperature (T) values.

Sample	T	κ_tot_	S	σ	PF	ZT
	(K)	(W/(m·K))	(µV/K)	(S/m)	(µW/(K^2^·cm))	
Hydro-Bi_2_Te_3_	298	0.92	−138	126,421	24.22	0.8
	373	0.9	−149	96,048	21.31	0.88
	473	1.16	−142	66,600	13.36	0.54
Polyol-Bi_2_Te_3_	298	0.95	−125	106,356	16.51	0.52
	373	0.86	−148	92,964	20.36	0.88
	473	0.9	−162	75,802	19.84	1.03

## Supplementary Materials

The following are available online at https://www.mdpi.com/article/10.3390/nano11082053/s1, Figure S1: Schematic of spark plasma sintering (SPS) process, sample geometry for thermal diffusivity, using Laser Flash technique, and power factor measurements. Figure S2: Rietveld refinement for Hydro- Bi_2_Te_3_ sample after SPS sintering, Figure S3: X-ray powder diffraction (XRPD) patterns showing a close up of the peaks with Miller indices of (006) and (015), Figure S4: Lorenz Number, Lo, estimated for Bi_2_Te_3_ samples using parabolic band model, Table S1: Survey of the synthesis methods, reaction conditions, morphology and size of Bi_2_Te_3_.
