# Supplementary material for: Minute-Made, High-Efficiency Nanostructured Bi2Te3 via High-Throughput Green Solution Chemical Synthesis
Source: Nanomaterials (Basel). 2021 Aug 12;11(8):2053. doi: 10.3390/nano11082053 (PMC8400796; doi:10.3390/nano11082053)
Supplement: Supplementary file 1 [file nanomaterials-11-02053-s001.zip › nanomaterials-1325711-supplementary.pdf]

Supplementary Material
for

Minute-made, high efficiency nanostructured Bi_2Te_3 via high-throughput green solution chemical synthesis

Bejan Hamawandi[1*], Hazal Batili[1], Moon Paul[1], Sedat Ballikaya[2], Nuzhet I. Kilic[1], Rafal Szukiewicz[3], Maciej Kuchowicz[3], Mats Johnsson[4], Muhammet S. Toprak[1*]

[1] Department of Applied Physics, KTH Royal Institute of Technology, 10691 Stockholm, Sweden

[2] Department of Physics, University of Istanbul, Istanbul, 34135, Turkey

[3] Institute of Experimental Physics, University of Wroclaw, Maxa Borna 9, 50-204 Wroclaw, Poland

[4] Department of Materials and Environmental Chemistry, Stockholm University, SE-106 91 Stockholm, Sweden

*bejan@kth.se *toprak@kth.se

Table S1. Survey of the synthesis methods, reaction conditions, morphology and size of Bi_2Te_3

Composition	Morphology	Synthesis Method	Temperature and Time	Dimensions	Reference
Bi_2Te_3	Nanotubes	Ultrasonic-assisted hydrothermal	180°C,48 h	50-1000 nm	[1]
Ag, Sb, Sn Doped Bi_2Te_3	Nanoparticles	Ultrasonic-assisted hydrothermal	200°C, 3 h	28-40 nm	[2]
Bi_2Te_3	Nanoparticles	Ultrasonic-assisted hydrothermal	70°C, 16 h, 20 h and 40 h	10-20 nm	[3]
Bi_2Te_3	Thin film	MOCVD	450°C	-	[4]
Bi_2Te_3/Sb_2Te_3	2D superlattice and thin films	MOCVD	350°C	-	[5]
Bi_2Te_3	Nanowires and nanoribbons	VLS	275 - 290°C	50nm-13µm	[6]
Bi_2Te_3	Nanoparticles	Microemulsion	-	10 nm	[7]
Bi_2Te_3	Nanoparticles and nanorods	Refluxing hydrothermal	100°C, 2-24 h	15-100 nm	[8]
Bi_2Te_3	Nanowires	Electrodeposition	RT	40-250 nm	[9]
Bi_2Te_3	Nanoparticles	Co-precipitation	RT	60-90 nm	[10]
Bi_2Te_3	Nanoparticles	co-precipitation	400°C 2h	80 nm	[11]
Bi_2Te_3	Nanosheets, nanorags, nano sheet-rod and nanorods	Solvothermal DMF, pyridine, acetone, ethano	100–180°C, 10–50 h	10 nm-10 µm	[12]

Composition	Morphology	Synthesis Method	Temperature and Time	Dimensions	Reference
Bi_2Te_3	Nanoparticles and nanowire	Solvothermal DMF	150°C, 24 h	15 nm-1μm	[13]
Bi_2Te_3	Nanoparticles and nanorod	Solvothermal DMF	100–180°C, 10–50 h	10-150 nm	[14]
Bi_2Te_3	Nanoparticles and nanoplates	Solvothermal Water, ethylene glycol	60°C, 120°C and 180°C, 16 h	30 nm	[15]
Bi_2Te_3	Nanoplates	Thermolysis Oleic acid	210°C, 24 h	30-200 nm	[16]
Bi_2Te_3	Nanoflower	Solvothermal water and ethanol	180°C, 8 h	30 nm-2 μm	[17]
Bi_2Te_3	Nanoparticles	Hydrothermal	100°C, 48 h	10 nm	[18]
Bi_2Te_3	Nanotubes	Hydrothermal	150°C, 24 h	30 nm-1μm	[19]
Bi_2Te_3	Nanosheets and nanoplates	Hydrothermal	120°C, 12 h	50-500 nm	[20]
Bi_2Te_3	Nanowires	Hydrothermal	65°C, 48 h	15 nm-1μm	[21]
Bi_2Te_3	Nanoplates, nanorods, and nanotubes	Hydrothermal	150°C, 180°C and 210°C, 24 h	20 nm-2 μm	[22]
Bi_2Te_3	Nanorods and nanoflakes	MW-assisted polyol method	0.5–6 h	20-400 nm	[23]
$Bi_{0.5}Sb_{1.5}Te_3$	Nanoplates	Hydrothermal	150°C, 24 h	30 nm- μm	[24]
Bi_2Te_3/ Sb_2Te_3	Nanoplates	Refluxing Water , ammonia	90°C, 18 h	13-80 nm	[25]
Bi_2Te_3	Nanowire	Hydrothermal	65°C for 72 h	Diameter: 15–20 nm Length: 1 μm	[26]
Bi_2Te_3	Nanorods and nanoflakes	MW-assisted polyol method	0.5–6 h	NR Width:20–50 nm NR length: 200–400 nm NF length: 90 to 150 nm	[23]
$Bi_{0.5}Sb_{1.5}Te_3$	Nanoplates	Hydrothermal	150°C, 24 h	Length: 100nm - μm Thickness: 30 nm to 100 nm	[24]
$Bi_{2-x}Sb_xTe_3$	Nanoplates	MW-assisted Polyol method	220 °C, 2 mins	Length: 50 - 500 nm Thickness: 50 nm	[27]

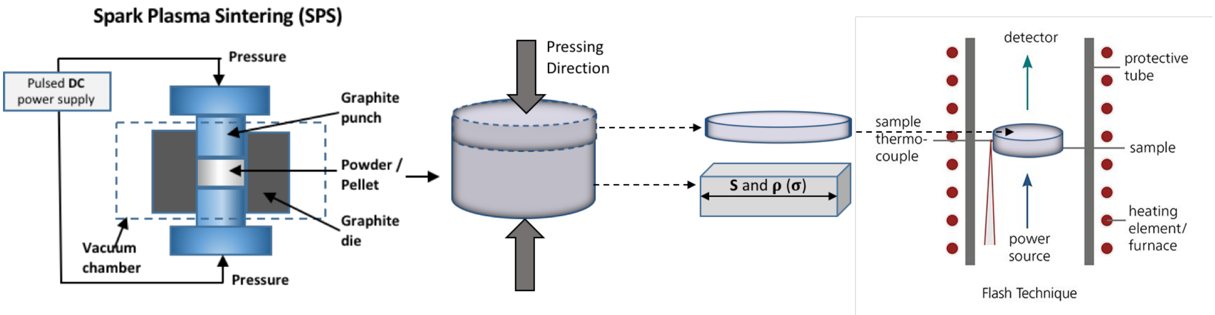

Figure S1: Schematic of Spark Plasma Sintering (SPS) process, sample geometry for thermal diffusivity, using Laser Flash technique, and power factor measurements.

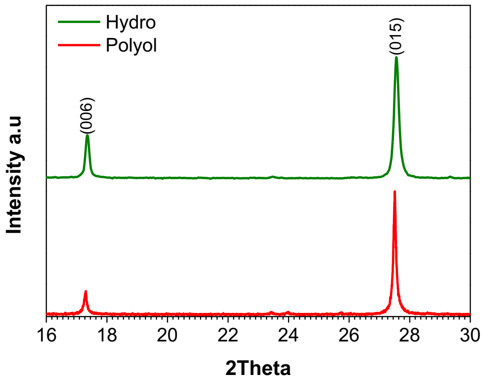

Figure S2: XRPD patterns showing a close up of the peaks with Miller indices of (006) and (015) for both the samples after SPS process. The relative peak intensity ratio (I_{006} / I_{015}) is a good indicative of texturing within the material, and was estimated as 0.38 for the Hydro-Bi_2Te_3 sample, and 0.19 for the Polyol-Bi_2Te_3 sample.

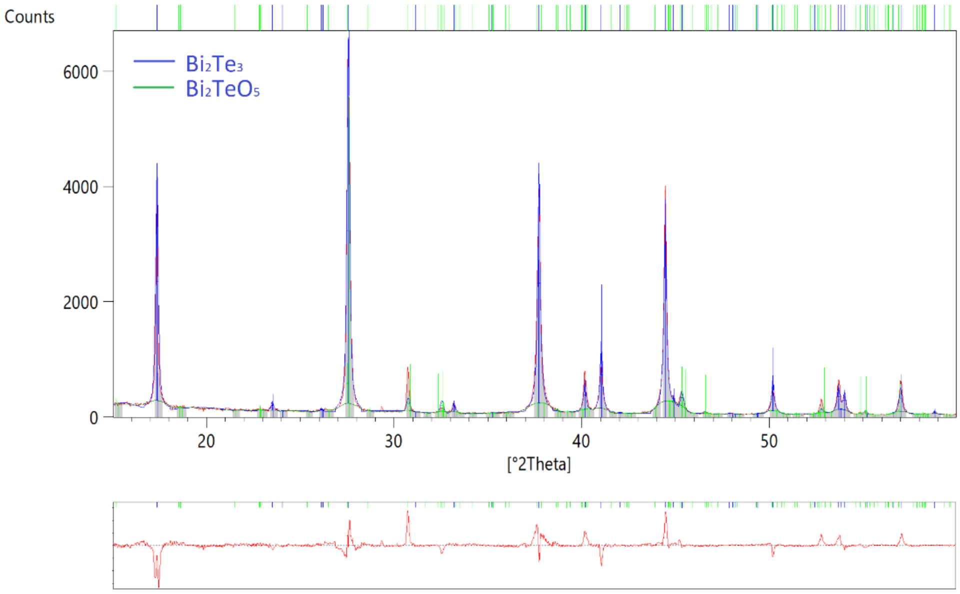

Figure S3: Rietveld refinement for Hydro-Bi_2Te_3 sample after SPS sintering. Quantification based on the Rietveld refinement yielded about 5.8% Bi_2TeO_5 phase.

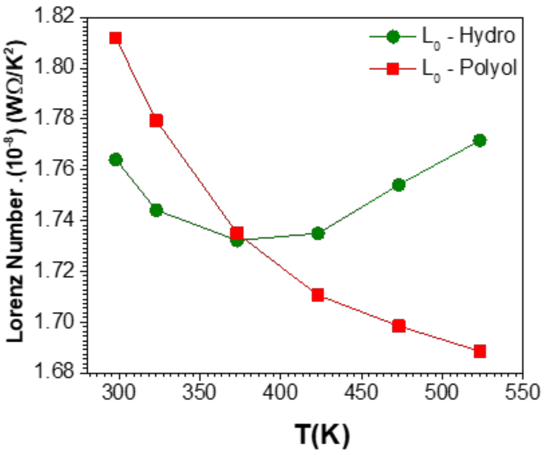

Figure S4: Lorenz Number, Lo, estimated for Bi_2Te_3 samples using parabolic band model.

References

1. Wang, Z.; Wang, F. qiang; Chen, H.; Zhu, L.; Yu, H. jun; Jian, X. yu Synthesis and characterization of Bi2Te3 nanotubes by a hydrothermal method. *J. Alloys Compd.* **2010**, *492*, 50–53, doi:10.1016/j.jallcom.2009.11.155.
2. Novaconi, S.; Vlazan, P.; Malaescu, I.; Badea, I.; Grozescu, I.; Sfirloaga, P. Doped Bi2Te3 nano-structured semiconductors obtained by ultrasonically assisted hydrothermal method. *Cent. Eur. J. Chem.* **2013**, *11*, 1599–1605, doi:10.2478/s11532-013-0291-7.
3. Zheng, Y.Y.; Zhu, T.J.; Zhao, X.B.; Tu, J.P.; Cao, G.S. Sonochemical synthesis of nanocrystalline Bi2Te3 thermoelectric compounds. *Mater. Lett.* **2005**, *59*, 2886–2888, doi:10.1016/j.matlet.2005.04.035.
4. Giani, A.; Pascal-Delannoy, F.; Boyer, A.; Foucaran, A.; Gschwind, M.; Ancey, P. Elaboration of Bi2Te3 by metal organic chemical vapor deposition. *Thin Solid Films* **1997**, *303*, 1–3, doi:10.1016/S0040-6090(97)00089-8.
5. Venkatasubramanian, R.; Colpitts, T.; Watko, E.; Lamvik, M.; El-Masry, N. MOCVD of Bi2Te3, Sb2Te3 and their superlattice structures for thin-film thermoelectric applications. *J. Cryst. Growth* **1997**, *170*, 817–821, doi:10.1016/S0022-0248(96)00656-2.
6. Lee, J.S.; Brittman, S.; Yu, D.; Park, H. Vapor-Liquid-Solid and Vapor-Solid Growth of Phase-Change Sb 2 Te 3 Nanowires and Sb 2 Te 3 / GeTe Nanowire Heterostructures ”. **2008**, 1–8.
7. Foos, E.E.; Stroud, R.M.; Berry, A.D. Synthesis and Characterization of Nanocrystalline Bismuth Telluride. *Nano Lett.* **2001**, *1*, 693–695, doi:10.1021/nl0156179.
8. Gupta, S.; Neeleshwar, S.; Kumar, V.; Chen, Y.Y. Synthesis of bismuth telluride nanostructures by refluxing method. *Adv. Mater. Lett.* **2012**, *3*, 50–54, doi:10.5185/amlett.2011.7285.
9. Sapp, S.A.; Lakshmi, B.B.; Martin, C.R. Template synthesis of bismuth telluride nano wires. *Adv. Mater.* **1999**, *11*, 402–404, doi:10.1002/(SICI)1521-4095(199903)11:5<402::AID-

ADMA402>3.0.CO;2-L.
10. Dhak, D.; Pramanik, P. Characterization of nanocrystalline bismuth telluride (Bi 2Te3) synthesized by a novel approach through aqueous precursor method. *J. Am. Ceram. Soc.* **2006**, *89*, 534–537, doi:10.1111/j.1551-2916.2005.00784.x.
11. Saleemi, M.; Toprak, M.S.; Li, S.; Johnsson, M.; Muhammed, M. Synthesis, processing, and thermoelectric properties of bulk nanostructured bismuth telluride (Bi 2Te 3). *J. Mater. Chem.* **2012**, *22*, 725–730, doi:10.1039/c1jm13880d.
12. Zhao, X.B.; Ji, X.H.; Zhang, Y.H.; Lu, B.H. Effect of solvent on the microstructures of nanostructured Bi 2Te3 prepared by solvothermal synthesis. *J. Alloys Compd.* **2004**, *368*, 349–352, doi:10.1016/j.jallcom.2003.08.070.
13. Deng, Y.; Wei, G.; Liu, J.; Zhou, X.; Wu, J.; Nan, C. Solvothermal preparation and characterization of nanocrystalline SnTe powder with different morphologies. *Xiyou Jinshu Cailiao Yu Gongcheng/Rare Met. Mater. Eng.* **2002**, *31*, 42.
14. Deng, Y.; Nan, C.W.; Wei, G.D.; Guo, L.; Lin, Y.H. Organic-assisted growth of bismuth telluride nanocrystals. *Chem. Phys. Lett.* **2003**, *374*, 410–415, doi:10.1016/S0009-2614(03)00783-8.
15. Jin, R.; Liu, J.; Li, G. Facile solvothermal synthesis, growth mechanism and thermoelectric property of flower-like Bi2Te3. *Cryst. Res. Technol.* **2014**, *49*, 460–466, doi:10.1002/crat.201400012.
16. Stavila, V.; Robinson, D.B.; Hekmaty, M.A.; Nishimoto, R.; Medlin, D.L.; Zhu, S.; Tritt, T.M.; Sharma, P.A. Wet-chemical synthesis and consolidation of stoichiometric bismuth telluride nanoparticles for improving the thermoelectric figure-of-merit. *ACS Appl. Mater. Interfaces* **2013**, *5*, 6678–6686, doi:10.1021/am401444w.
17. Xu, Y.; Ren, Z.; Cao, G.; Ren, W.; Deng, K.; Zhong, Y. Fabrication and characterization of Bi2Te3 nanoplates via a simple solvothermal process. *Phys. B Condens. Matter* **2009**, *404*, 4029–4033, doi:10.1016/j.physb.2009.07.153.
18. Kim, C.; Kim, D.H.; Han, Y.S.; Chung, J.S.; Park, S.H.; Kim, H. Fabrication of bismuth telluride nanoparticles using a chemical synthetic process and their thermoelectric evaluations. *Powder Technol.* **2011**, *214*, 463–468, doi:10.1016/j.powtec.2011.08.049.
19. Giri, L.; Mallick, G.; Jackson, A.C.; Griep, M.H.; Karna, S.P. Synthesis and characterization of high-purity, single phase hexagonal Bi2Te3 nanostructures. *RSC Adv.* **2015**, *5*, 24930–24935, doi:10.1039/c5ra02303c.
20. Xu, Y.; Ren, Z.; Ren, W.; Cao, G.; Deng, K.; Zhong, Y. Hydrothermal synthesis of single-crystalline Bi2Te3 nanoplates. *Mater. Lett.* **2008**, *62*, 4273–4276, doi:10.1016/j.matlet.2008.06.055.
21. Sun, T.; Zhao, X.B.; Zhu, T.J.; Tu, J.P. Aqueous chemical reduction synthesis of Bi2Te3 nanowires with surfactant assistance. *Mater. Lett.* **2006**, *60*, 2534–2537, doi:10.1016/j.matlet.2006.01.033.
22. Kim, H.J.; Han, M.; Kim, H.; Lee, W.; Kim, S. Morphology Controlled Synthesis of Nanostructured Bi 2 Te 3. **2012**, *33*, 3977–3980.
23. Zhou, B.; Zhao, Y.; Pu, L.; Zhu, J.J. Microwave-assisted synthesis of nanocrystalline Bi2Te 3. *Mater. Chem. Phys.* **2006**, *96*, 192–196, doi:10.1016/j.matchemphys.2005.07.010.
24. Zhang, Y.; Xu, G.; Mi, J.; Han, F.; Wang, Z.; Ge, C. Hydrothermal synthesis and thermoelectric properties of nanostructured Bi0.5Sb1.5Te3 compounds. *Mater. Res. Bull.* **2011**, *46*, 760–764, doi:10.1016/j.materresbull.2010.11.024.
25. Pelz, U.; Kaspar, K.; Schmidt, S.; Dold, M.; Jägle, M.; Pfaadt, A.; Hillebrecht, H. An aqueous-chemistry approach to nano-bismuth telluride and nano-antimony telluride as thermoelectric materials. *J. Electron. Mater.* **2012**, *41*, 1851–1857, doi:10.1007/s11664-012-2099-1.

26. Cao, Y.Q.; Zhu, T.J.; Zhao, X.B. Thermoelectric Bi2Te3 nanotubes synthesized by low-temperature aqueous chemical method. *J. Alloys Compd.* **2008**, *449*, 109–112, doi:10.1016/j.jallcom.2006.01.116.
27. Hamawandi, B.; Ballikaya, S.; Batili, H.; Roosmark, V.; Orlovská, M.; Yusuf, A.; Johnsson, M.; Szukiewicz, R.; Kuchowicz, M.; Toprak, M.S. Facile solution synthesis, processing and characterization of n-and p-type binary and ternary Bi-Sb tellurides. *Appl. Sci.* **2020**, *10*, doi:10.3390/app10031178.
